# Expression of phosphoenolpyruvate carboxykinase linked to chemoradiation susceptibility of human colon cancer cells

**DOI:** 10.1186/1471-2407-14-160

**Published:** 2014-03-06

**Authors:** Ji-Won Park, Seung Cheol Kim, Won Ki Kim, Jun Pyu Hong, Kyung-Hee Kim, Hyun Yang Yeo, Jae Yong Lee, M Sun Kim, Jong Heon Kim, Se Young Yang, Dae Yong Kim, Jae Hwan Oh, Jae Youl Cho, Byong Chul Yoo

**Affiliations:** 1 Colorectal Cancer Branch, Research Institute, National Cancer Center, Goyang, Gyeonggi, Republic of Korea; 2 Center for Colorectal Cancer, Hospital, National Cancer Center, Goyang, Gyeonggi, Republic of Korea; 3 Division of Gynecologic Oncology, Department of Obstetrics and Gynecology, Ewha Womans University Mokdong Hospital, College of Medicine, Ewha Womans University, Seoul, Republic of Korea; 4 Cancer Cell and Molecular Biology Branch, Research Institute, National Cancer Center, Goyang, Gyeonggi, Republic of Korea; 5 Department of Genetic Engineering, Sungkyunkwan University, Suwon, Gyeonggi, Republic of Korea

**Keywords:** mPEPCK, 5-FU resistance, Colon cancer, Chemoradiotherapy, Prediction

## Abstract

**Background:**

Resistance to 5-fluorouracil (5-FU) in patients with colorectal cancer prevents effective treatment and leads to unnecessary and burdensome chemotherapy. Therefore, prediction of 5-FU resistance is imperative.

**Methods:**

To identify the proteins linked to 5-FU resistance, two-dimensional gel electrophoresis-based proteomics was performed using the human colon cancer cell line SNU-C4R with induced 5-FU resistance. Proteins showing altered expression in SNU-C4R were identified by matrix-associated laser desorption/ionization–time-of-flight analysis, and their roles in susceptibility to 5-FU or radiation were evaluated in various cell lines by transfection of specific siRNA or creation of overexpression constructs. Changes in cellular signaling and expression of mitochondrial apoptotic factors were investigated by Western Blot analysis. A mitochondrial membrane potential probe (JC-1 dye) and a flow cytometry system were employed to determine the mitochondrial membrane potential. Finally, protein levels were determined by Western Blot analysis in tissues from 122 patients with rectal cancer to clarify whether each identified protein is a useful predictor of a chemoradiation response.

**Results:**

We identified mitochondrial phosphoenolpyruvate carboxykinase (mPEPCK) as a candidate predictor of 5-FU resistance. PEPCK was downregulated in SNU-C4R compared with its parent cell line SNU-C4. Overexpression of mPEPCK did not significantly alter the susceptibility to either 5-FU or radiation. Suppression of mPEPCK led to a decrease in both the cellular level of phosphoenolpyruvate and the susceptibility to 5-FU and radiation. Furthermore, the cellular levels of phosphoenolpyruvate (an end product of PEPCK and a substrate of pyruvate kinase), phosphorylated AKT, and phosphorylated 4EBP1 were decreased significantly secondary to the mPEPCK suppression in SNU-C4. However, mPEPCK siRNA transfection induced changes in neither the mitochondrial membrane potential nor the expression levels of mitochondrial apoptotic factors such as Bax, Bcl-2, and Bad. Downregulation of total PEPCK was observed in tissues from patients with rectal cancer who displayed poor responses to preoperative 5-FU-based radiation therapy.

**Conclusion:**

Our overall results demonstrate that mPEPCK is a useful predictor of a response to chemoradiotherapy in patients with rectal cancer.

## Background

The use of 5-fluorouracil (5-FU) as a chemotherapeutic agent for colorectal cancer (CRC) is pervasive in the field of medicine because of its ability to act as an S-phase-specific antimetabolite and to suppress cell proliferation [1,2]. In chemoradiotherapy (CRT), radiation is often performed in conjunction with 5-FU [3]. However, resistance to radiation has been reported in some patients with CRC, and their poor response to either 5-FU or radiation treatment makes preoperative CRT ineffective and burdensome. Activated NF-κB along with other NF-κB–regulated gene products, such as Bcl-xL, cyclin D1, matrix metalloproteinase 9, vascular endothelial growth factor, or COX-2, might contribute to radiation resistance by promoting prosurvival signaling [4]. Ashele et al. found that 5-FU resistance may be caused by impaired polyglutamylation of the thymidylate synthase cofactor CH_2_FH_4_ and decreased incorporation of 5-FU into RNA [5].

Phosphoenolpyruvate carboxykinase (PEPCK) is known to exist in both the cytosol and mitochondria in the mouse, human, and chicken. This enzyme catalyzes the reversible decarboxylation of oxaloacetic acid with the concomitant transfer of the gamma-phosphate of GTP to form phosphoenolpyruvate (PEP) and GDP [6,7]. Cytosolic PEPCK has been investigated extensively and is considered to be a key enzyme in gluconeogenesis and glyceroneogenesis [8,9]. In contrast, the mitochondrial isoform of PEPCK (mPEPCK) has a metabolic role that is complementary to but distinct from that of cytosolic PEPCK in the regulation of gluconeogenesis [9].

In this study, we found that mPEPCK is downregulated in the human colon cancer cell line SNU-C4R with induced 5-FU resistance. We herein present the results of this study and discuss how the total amount of PEPCK may affect the CRT response.

## Methods

### Human colon cancer cell lines and establishment of 5-FU–resistant cell lines

The human colon cancer cell lines SNU-C4, SNU-C5, SNU-81, SNU-407, DLD-1, SW620, LoVo, HCT-116, NCI-H747, NCI-H508, and CaCo2 were obtained from the Korean Cell Line Bank (Seoul, Korea). The cell line SNU-C4R, which is resistant to the anticancer agent 5-FU (Choongwae Pharma Corporation, Gyeonggi, Korea), was derived from SNU-C4 as described previously [10].

### Tissue from patients with CRC

All patients included in this study provided written informed consent to participate. The study protocol was approved by the Institutional Review Board of the National Cancer Center. In total, 122 patients with locally advanced mid/low rectal cancer who had undergone preoperative CRT and radical proctectomy at the National Cancer Center of Korea were enrolled in this study (Table 1). All patients underwent a staging workup before preoperative CRT. Tumor tissues had been obtained by colonoscopic biopsy prior to treatment. As described in previous studies, radiotherapy was delivered to the whole pelvis at a dose of 45 Gy in 25 fractions followed by a 5.4-Gy boost in 3 fractions within 6 weeks (n = 103) or a dose of 25 Gy over 5 consecutive days using helical tomotherapy (TomoTherapy, Inc., Madison, WI) (n = 19) [11,12]. Concurrent chemotherapy was administered to all patients in the form of a fluoropyrimidine-based chemotherapeutic regimen: 5-fluorouracil and leucovorin (n = 84; one or two cycles of 5-FU [400 mg/m^2^] and leucovorin [20 mg/m^2^] during RT), capecitabine (n = 6; oral administration of capecitabine [825 mg/m^2^] twice daily during RT without weekend breaks), or uracil-tegafur (n = 32; oral administration of uracil-tegafur [400 mg/m^2^/day] and leucovorin [90 mg/day] 5 days per week during radiation). All patients underwent total mesorectal excision 4 to 8 weeks after the completion of preoperative CRT. The pathological tumor stage was determined according to the 6th TNM classification system recommended by the American Joint Committee on Cancer [13]. The CRT response was evaluated using the tumor regression grade system proposed by Dworak et al. [14]. The tumor regression grades were defined as follows: grade 0, no regression; grade 1, dominant tumor mass with obvious fibrosis and/or vasculopathy (minimal regression); grade 2, dominant fibrotic changes with obvious tumor cells or groups of cells (moderate regression); grade 3, fibrotic tissue with or without mucous substance containing very few tumor cells and that are difficult to detect microscopically (near-complete regression); and grade 4, fibrotic mass or acellular mucin pools only, without detectable tumor cells (complete regression). Tumors with grade 3 or 4 regression comprised the “good response” group. Other tumors comprised the “poor response” group.

**Table 1:** Characteristics of patients with rectal cancer

**Characteristics**	**Patients number (%)**
Sex	
Male	81 (66.4)
Female	41 (33.6)
Age (years), median (range)	59 (29-84)
ECOG PS	
0	113 (92.6)
1	8 (6.6)
2	1 (0.8)
Tumor distance from anal verge (cm), median (range)	6 (0-9)
Pretreatment CEA (ng/mL), median (range)	4 (0.5-206)
Histologic grade	
Well differentiated	29 (23.8)
Moderately differentiated	91 (74.6)
Poorly differentiated	1 (0.8)
Unknown	1 (0.8)
Tumor size (cm), median (range)	2.5 (0-7.2)
Dworak grade	
1	27 (22.1)
2	76 (62.3)
3	12 (9.8)
4	7 (5.7)
T classification	
0	10 (8.2)
is	2 (1.6)
1	3 (2.5)
2	28 (23.0)
3	71 (58.2)
4	8 (6.5)
N classification	
0	71 (58.2)
1	33 (27.0)
2	18 (14.8)

ECOG PS: indicates Eastern Collaborative Oncology Group Performance Status.

### MTT assay

A colorimetric assay using the tetrazolium salt MTT (3-[4,5-dimethylthiazol-2-yl]-2,5-diphenyltetrazolium bromide) was used to assess suppression of cell survival by 5-FU (Dong-A Pharmaceutical Co., Ltd., Seoul, Korea) and irradiation. Cells grown in a 96-well plate were incubated with 5-FU and/or irradiated in a self-contained X-ray system (X-RAD 320; Precision X-Ray, Inc., North Branford, CT, USA) at 290 kV and 10 mA. The dose rate was 8 Gy/min. Single-cell suspensions were prepared, and the cell density was measured. MTT assays were performed as described previously [10]. All experiments were performed three times, and the mean and standard deviation of the half-maximal inhibitory concentration (IC_50_, μg/ml) were calculated.

### Two-dimensional gel electrophoresis-based comparative proteomics

Two-dimensional gel electrophoresis (2-DE) analysis was performed as described previously [10]. A 0.15-mg protein sample was applied to 13-cm immobilized nonlinear gradient strips (pH 3–10), focused at 8000 V within 3 h, and separated in 10% polyacrylamide gels (Serva, Heidelberg, Germany; Bio-Rad, Hercules, CA). The 2-DE gels were stained with Colloidal Coomassie Blue (Invitrogen, Carlsbad, CA) for 24 h and then destained with deionized water. Proteins showing abnormal expression were subjected to matrix-associated laser desorption/ionization–mass spectroscopy (MALDI-MS) analysis for identification. MALDI-MS analysis of 2-DE protein spots was performed as previously described [10]. Mass spectra were first calibrated in the closed external mode using the 4700 Proteomics Analyzer Calibration Mixture (AB SCIEX, Foster City, CA) and analyzed with GPS Explorer software, version 3.5 (AB SCIEX). The acquired MS/MS spectra were searched against the Swiss-Prot and NCBI databases by an in-house version of MASCOT.

### Whole-protein extraction and subcellular fractionation

Cells or tissues were homogenized in four volumes of cell lysis buffer (Pro-Prep; iNtRON Biotechnology, Gyeonggi, Republic of Korea) using a Sample Grinding Kit (GE Healthcare, Piscataway, NJ). The total homogenate was incubated on ice for 20 min and centrifuged at 600 × *g* for 5 min. The supernatant was used as a whole protein extract. The cytosolic fraction was obtained by centrifugation of the whole protein extract at 11,000 × *g* for 10 min.

For isolation of an enriched, functional mitochondrial fraction from cells, the Mitochondria Isolation Kit (Catalog No. MITISO1; Sigma, Saint Louis, MO) was used as recommended by the manufacturer. Briefly, cells were suspended with 10 volumes of the extraction buffer (20 mM MOPS, pH 7.5, containing 110 mM KCl, 1 mM EGTA, and 0.25 mg/ml trypsin) and incubated on ice for 3 min. The cells were then centrifuged for a few seconds. The supernatant was removed by aspiration, and eight volumes of the extraction buffer were added. After incubation on ice for 20 min, the albumin solution was added to a final concentration of 10 mg/ml to quench the proteolytic reaction. The solution was then centrifuged for a few seconds. The supernatant was removed by aspiration, and the pellet was washed with 8 volumes of the extraction buffer. This step was repeated. The pellet was then homogenized and centrifuged at 600 × *g* for 5 min. The supernatant liquid was transferred to a new tube and centrifuged at 11,000 × *g* for 10 min. The pellet was suspended in the storage buffer (10 mM HEPES, pH 7.4, containing 250 mM sucrose, 1 mM ATP, 0.08 mM ADP, 5 mM sodium succinate, 2 mM K_2_HPO_4_, and 1 mM DTT [~40 ml per 100 mg of tissue]) and used as a mitochondrial fraction.

### Western blot analysis

Western Blot analysis was performed as described previously [10]. Supernatants of cell homogenates containing equivalent amounts of protein were subjected to SDS-PAGE and transferred to polyvinylidene fluoride membranes (Millipore, Bedford, MA). The membranes were incubated for 2 h at room temperature with primary anti-PEPCK antibody that attaches to both cytosolic and mitochondrial PEPCK) (Catalog No. sc-32879; Santa Cruz Biotechnology, Inc., Dallas, TX), VDAC (Abcam, Cambridge, UK), actin (Capital Bioscience, Rockville, MD), Bax (Abcam), Bcl-2 (Abcam), Bad (Abcam), AKT (Cell Signaling Technology, Inc., Danvers, MA), 4EBP1 (Cell Signaling Technology, Inc.), mTOR (Cell Signaling Technology, Inc.), p-AKT (Ser 473; Cell Signaling Technology, Inc.), p-mTOR (Ser 2448; Cell Signaling Technology, Inc.), or p-4EBP1 (Thr 70; Cell Signaling Technology, Inc.). The membranes were washed, incubated with diluted HRP-conjugated secondary antibody (SouthernBiotech, Birmingham, AL), and exposed to film (Blue XB-1; Kodak, Rochester, NY).

### Measurement of PEP

The level of intracellular PEP was measured using a PEP assay kit (BioVision Inc., Milpitas, CA) as recommended by the manufacturer.

### siRNA transfection

Transfection of mPEPCK siRNA (Santa Cruz Biotechnology, Santa Cruz, CA) and negative control siRNA (Qiagen, Chatsworth, CA) was performed with the HiPerFect transfection reagent (Qiagen, Hilden, Germany) according to the manufacturer’s protocol.

### mPEPCK expression construct

To generate pEGFPc1-mPEPCK, a human fetal liver cDNA library (Clontech, Mountain View, CA) was PCR-amplified with the following oligomers specific to human mPEPCK (Macrogen, Seoul, Korea): sense, 5′-GGAATTCCATGGCCGCATTGTACCGCC-3′ and antisense, 5′-CGGGATCCTCAGGTCACATTTTGTGCACACGTC-3′. The amplified DNA was digested with *EcoR*I-*BamH*I and then inserted into pEGFPc1 (Clontech). The construct was verified by DNA sequencing (Cosmo Genetech, Seoul, Korea).

### Detection of mitochondrial membrane potential

Cells were treated with 5-FU and radiation as described above, then dislodged by trypsin. After centrifugation, the pelleted cells were washed with PBS and suspended in 5 μg/ml of 5,5′,6,6′-tetrachloro-1,1′,3,3′-tetraethylbenzimidazolylcarbocyanine iodide (JC-1 dye; Molecular Probes, Eugene, OR) for 15 min at 37°C in an incubation chamber. The cells were washed again with PBS and then resuspended in PBS. The stained cells were analyzed for their relative apoptosis content using a flow cytometry system (FACSCalibur; BD Biosciences, Franklin Lakes, NJ).

### Statistical analyses

Between-group differences were calculated using Student’s *t*-test, and within-group correlations were calculated using Spearman’s rank-coefficient. Statistical significance was set at *P* < 0.05.

## Results

### Downregulated mPEPCK in human colon cancer cell line SNU-C4R with induced 5-FU resistance and its correlation with 5-FU response

**Figure 1:**
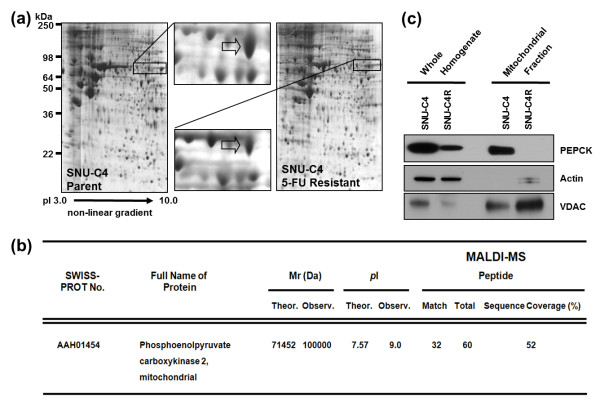
**Downregulated mitochondrial phosphoenolpyruvate carboxykinase (mPEPCK) in the human colon cancer cell line SNU-C4R with induced 5-FU resistance. (a)** Selection of downregulated protein spot on 2-DE gel images. By comparing the 2-DE gel images, a ~100-kDa protein was identified as being downregulated in SNU-C4R. **(b)** Identification of mPEPCK by MALDI-MS analysis. The selected protein spot was identified as mPEPCK. **(c)** Western Blot analysis for confirmation of downregulation of mPEPCK in SNU-C4R. Total PEPCK (cytosolic and mitochondrial isoforms) and mPEPCK expression was significantly lower in SNU-C4R than in its parent cell line SNU-C4.

We performed 2-DE to investigate dysregulated protein expression in the human colon cancer cell line SNU-C4R with induced 5-FU resistance. In 2-DE gel image analysis, a ~100-kDa protein was identified as being downregulated in SNU-C4R (Figure 1a). This protein spot was identified as mPEPCK through MALDI-MS analysis and confirmed by Western Blot (Figure 1b and c).

**Figure 2:**
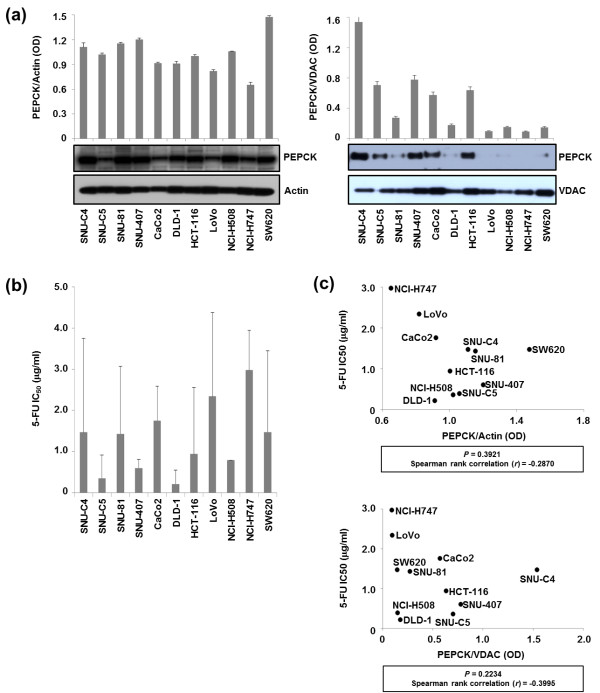
**Quantification of PEPCK expression levels in 11 human colon cancer lines with different responses to 5-FU. (a)** The expression levels of PEPCK in whole homogenates and mitochondrial fractions of different cell lines were normalized with actin and VDAC, respectively. **(b)** Differential responses of cell lines to 5-FU. The 5-FU IC_50_ was determined in 11 colon cancer cell lines 96 h after 5-FU treatment by MTT assay. **(c)** No correlation was observed between mPEPCK expression and the responses of cell lines to 5-FU. The 5-FU IC_50_ values of the various cell lines were plotted against their PEPCK expression levels in whole homogenates (upper panel) and mitochondrial fractions (lower panel) normalized to actin and VDAC, respectively.

Differential PEPCK expression levels in the whole homogenates and mitochondrial fractions from 11 human colon cancer cell lines were detected by Western Blot and normalized to actin and VDAC, respectively (Figure 2a). As expected, the 5-FU IC_50_ (the concentration of 5-FU that results in a 50% decrease in cell survival compared with control) varied among CRC cell lines (Figure 2b). However, the PEPCK expression levels in neither the mitochondrial fractions nor the whole homogenates showed a correlation with 5-FU IC_50_ (Figure 2c).

### Decreased cellular level of PEP and reduced susceptibility to 5-FU/radiation induced by mPEPCK suppression in SNU-C4

**Figure 3:**
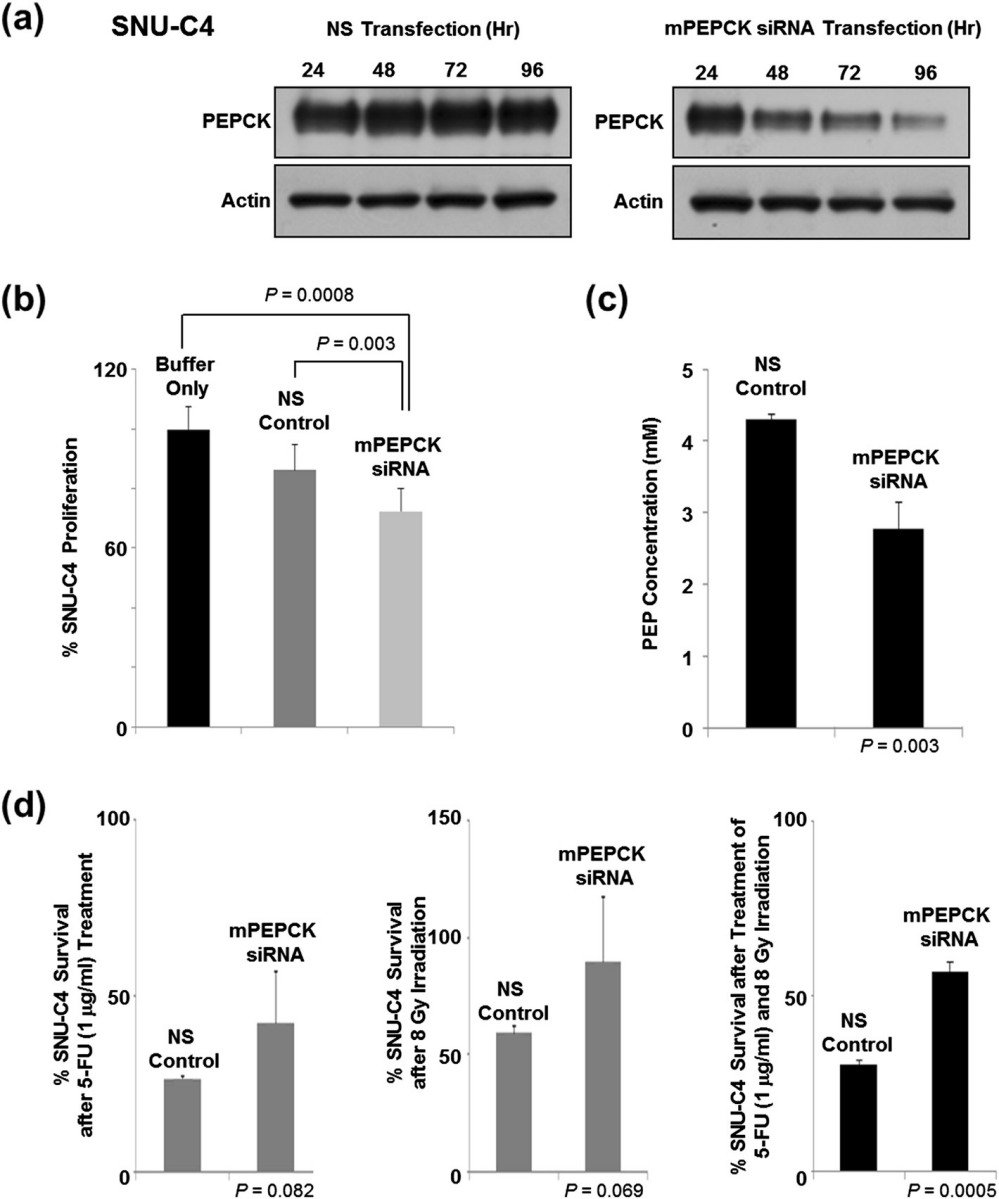
**Effect of mPEPCK downregulation on 5-FU response in SNU-C4 cells. (a)** Suppressed mPEPCK expression in SNU-C4 cells transfected with mPEPCK siRNA. The PEPCK expression levels decreased over time in the mPEPCK siRNA-transfected cells, while the nonsilenced (NS) control showed no apparent changes in the PEPCK expression levels. **(b)** Decreased cell proliferation rate after mPEPCK suppression. With no 5-FU or radiation treatment, artificial suppression of mPEPCK decreased the rate of cell proliferation. **(c)** Reduced cellular PEP level after mPEPCK suppression. **(d)** Increased 5-FU and radiation resistance was observed after mPEPCK suppression in SNU-C4 cells. MTT assay showed that the mPEPCK siRNA-transfected SNU-C4 cells exhibited higher survival rates than the control after combination treatment comprising 5-FU and radiation.

mPEPCK siRNA transfection was performed using SNU-C4 to investigate the role of mPEPCK in 5-FU resistance. At 96 h after siRNA transfection, significantly less PEPCK was detected in the mPEPCK-suppressed cells than in the nonsilenced (NS) control (Figure 3a). mPEPCK suppression itself reduced the proliferation rate of SNU-C4 without any 5-FU or radiation treatment (Figure 3b). Furthermore, the cellular level of PEP (an end product of PEPCK and substrate of pyruvate kinase) was also significantly decreased by the mPEPCK suppression in SNU-C4 (Figure 3c). The survival rates of the mPEPCK-suppressed SNU-4 cells after 1 μg/ml 5-FU treatment or 8-Gy radiation were higher than those of the control, but the difference did not reach statistical significance (*P* = 0.082 and 0.069, respectively) (Figure 3d). Suppression of mPEPCK significantly increased survival of SNU-4 cells after combined treatment comprising 5-FU and radiation (*P* = 0.0005) (Figure 3d).

**Figure 4:**
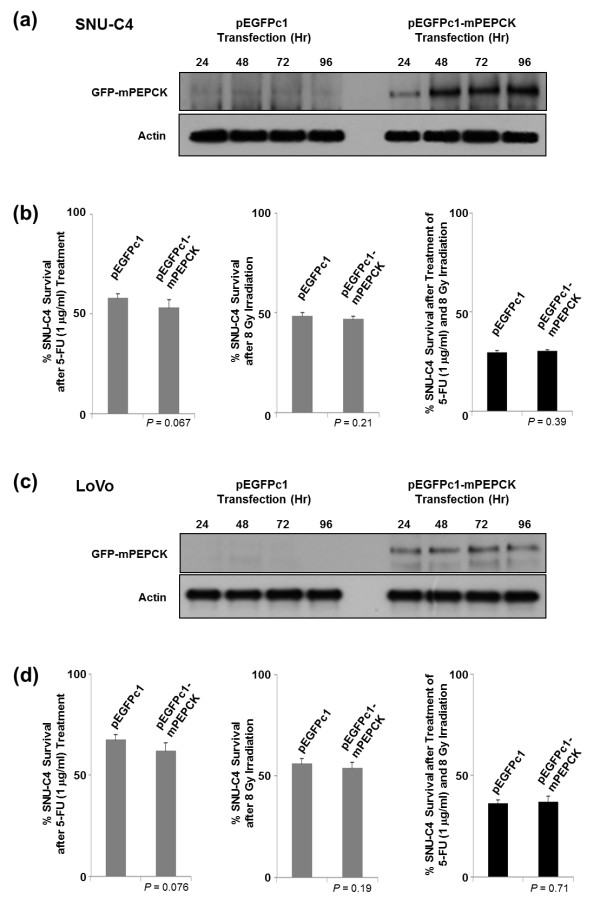
**Effect of mPEPCK overexpression on 5-FU response in SNU-C4 cells.** Overexpression of mPEPCK in **(a)** SNU-C4 and **(c)** LoVo transfected with pEGFPc1-mPEPCK. Upregulated mPEPCK was monitored over 24-, 48-, 72-, and 96-h time periods by Western Blot, while the pEGFPc1-transfected control showed no changes in PEPCK expression levels. No effect of overexpression of mPEPCK was observed in **(b)** SNU-C4 or **(d)** LoVo on the susceptibility to 5-FU, irradiation, or combination treatment comprising 5-FU and irradiation. Overexpression of mPEPCK slightly increased the susceptibility to 5-FU in both SNU-C4 and LoVo cells, but the difference was not statistically significant.

The effects of mPEPCK overexpression using pEGFPc1-mPEPCK vector were also investigated. Overexpressed mPEPCK was localized in the cytoplasm rather than in the mitochondria (data not shown). Overexpression of mPEPCK in SNU-C4 and LoVo showed slightly increased susceptibility to 5-FU (*P* = 0.067), but not to radiation or combined treatment (*P* = 0.21 and 0.39, respectively) (Figure 4). The response of LoVo to 5-FU/radiation after mPEPCK overexpression showed a pattern similar to that of SNU-C4 (Figure 4c and d).

### Effect of mPEPCK suppression on mitochondrial membrane potential and signal-transducing molecules

**Figure 5:**
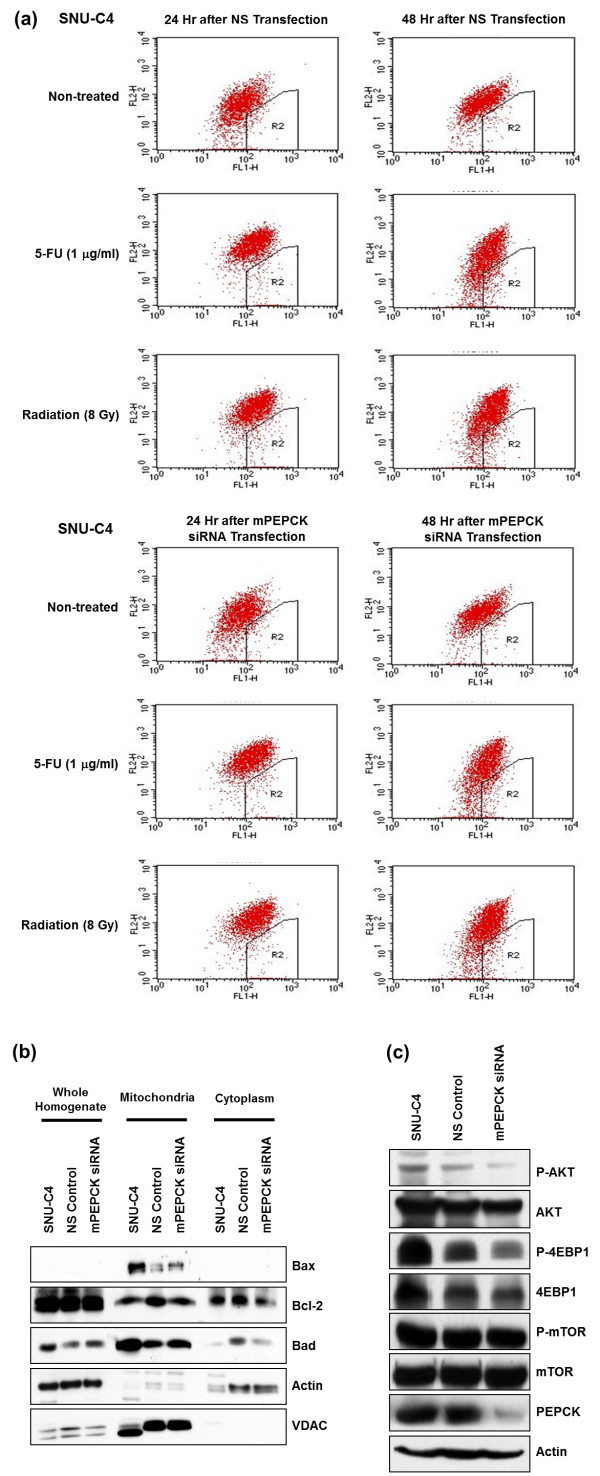
**Cellular changes in mPEPCK-suppressed SNU-C4 cells. (a)** No change in the mitochondrial membrane potential or apoptotic cell death was observed in SNU-C4 transfected with mPEPCK siRNA. Flow cytometry was performed using JC-1 to determine the mitochondrial membrane potential and apoptotic cell death rate. **(b)** No changes in apoptosis-related mitochondrial proteins were observed in the non-transfected, NS, or mPEPCK siRNA-transfected SNU-C4 cells. Whole cellular homogenate, mitochondrial, and cytosolic fractions were used for Western Blot analysis. Expression of Bax, Bcl-2, and Bad was not affected by mPEPCK suppression. **(c)** Altered phosphorylation level of main signaling molecules after mPEPCK suppression in SNU-C4 cells. Phosphorylation levels of AKT and 4EBP1 were significantly decreased in SNU-C4 cells after mPEPCK siRNA transfection.

To determine whether the decreased cell survival after mPEPCK suppression was caused by alteration of the mitochondrial membrane potential, JC-1 dye and the FACSCalibur flow cytometry system were employed to determine the mitochondrial membrane potential. However, neither 1 μg/ml 5-FU treatment nor 8-Gy radiation resulted in a significant difference between the mPEPCK siRNA-transfected cells and the control in terms of the mitochondrial membrane potential or level of apoptosis following the change in the mitochondrial potential (Figure 5a). Further Western Blot analysis revealed that the expression levels of mitochondrial apoptotic factors such as Bax, Bcl-2, and Bad also remained unchanged in the mPEPCK-suppressed cells (Figure 5b).

Examination of the phosphorylation level of signal-transducing molecules after mPEPCK siRNA transfection revealed lower levels of phosphorylated AKT (p-AKT) and phosphorylated 4EBP1 (p-4EBP1) in mPEPCK siRNA-transfected cells than in the NS control or normal, nontransfected SNU-C4 line (Figure 5c).

### Poor response to CRT in patients with rectal cancer is associated with low PEPCK expression

**Figure 6:**
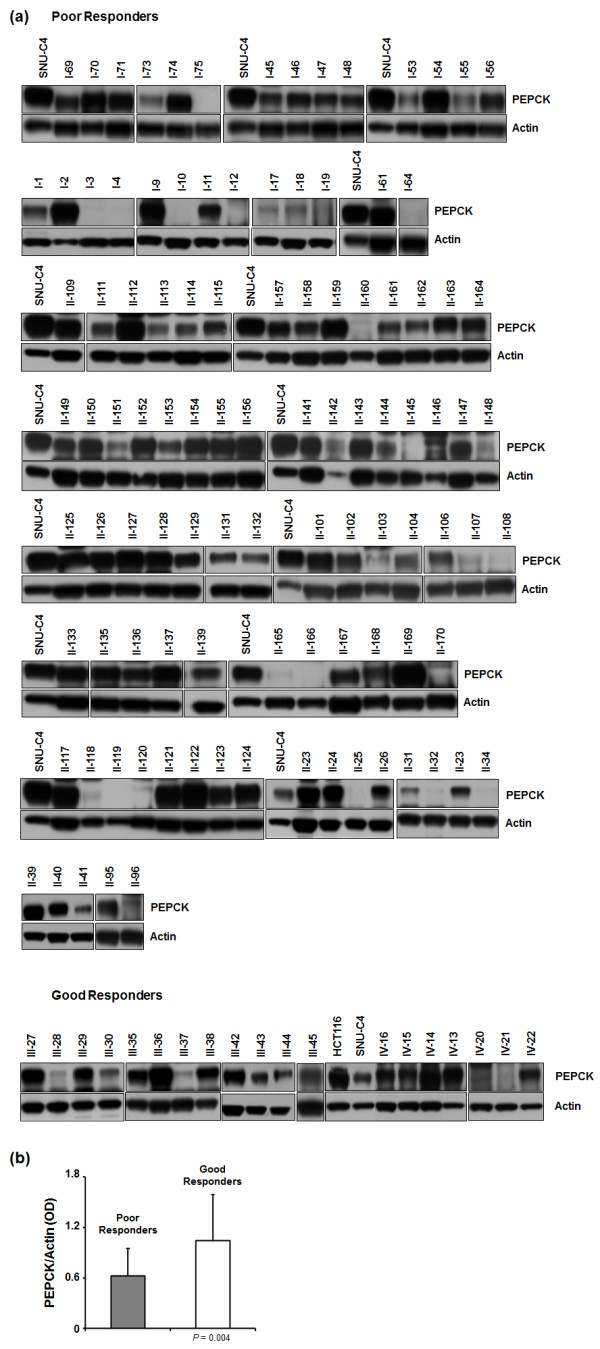
**PEPCK expression levels in patients with rectal cancer. (a)** Western Blot analysis was performed to determine the PEPCK expression levels in 122 patients with rectal cancer. The patients were divided into poor and good response groups according to their response to chemoradiotherapy (see Methods). The PEPCK expression levels in SNU-C4 and HCT-116 were used as controls. **(b)** PEPCK expression levels were linked to the chemoradiotherapy response. The PEPCK expression levels were normalized to the actin expression levels.

The expression levels of total PEPCK (cytoplasmic plus mitochondrial PEPCK) in 122 patients with rectal cancer were measured by Western Blot, and the optical densities of the spots were measured from scanned images (Figure 6a; Additional file 1: Table S1). The PEPCK expression levels of SNU-C4 and HCT-116 were used as controls. The levels of PEPCK expression were significantly lower in tumors with a poor response (tumor regression grade of 1 or 2) than in those with a good response (tumor regression grade of 3 or 4) (*P* = 0.004) (Figure 6b).

## Discussion

In this study, we investigated the possible role of mPEPCK as a predictor of 5-FU and radiation susceptibility in patients with CRC. Using different CRC cell lines and tumor tissues of patients with CRC who were treated with 5-FU-based CRT, we showed that mPEPCK was downregulated in 5-FU–resistant cells and tumor tissues of patients who were resistant to 5-FU–based CRT. Several mechanisms of reduced PEPCK expression have been proposed. Antioxidants can suppress PEPCK expression through the transcription factor activator protein 1, or PEPCK can be degraded by proteasome pathways in response to high glucose levels [15,16]. Although the cause of PEPCK downregulation in nonresponders to 5-FU–based CRT has not yet been elucidated, low PEPCK expression seems to be related to a poor response.

Downregulation of mPEPCK in the human colon cancer cell line SNU-C4R with induced 5-FU resistance was confirmed by 2-DE and MALDI-MS (Figure 1a and b). mPEPCK expression and responses to 5-FU differed among the 11 human colon cancer cell lines (Figure 2). However, there was no significant correlation between mPEPCK expression and 5-FU susceptibility in any of these 11 colon cancer cell lines (Figure 2c). This finding suggests that downregulation of mPEPCK may be linked to induced 5-FU resistance, not intrinsic 5-FU susceptibility, in colon cancer cells. mPEPCK was suppressed using mPEPCK siRNA to clarify the role of mPEPCK in the cellular response to 5-FU, and successful suppression was confirmed by the observation of decreasing PEPCK expression levels over time after siRNA transfection (Figure 3a). Compared with the NS control, the mPEPCK-suppressed SNU-C4 cells demonstrated slower proliferation (Figure 3b), but a higher survival rate, after combined treatment comprising 5-FU and radiation (*P* = 0.0005) (Figure 3d). These findings suggest the involvement of mPEPCK in response to both 5-FU and radiation. Slow growth may be caused by downregulated energy metabolism. Because PEP is the final product of PEPCK as well as the substrate of pyruvate kinase, which is an enzyme involved in a rate-limiting step of glycolysis, a low cellular level of PEP followed by mPEPCK suppression may lead to slow proliferation of colon cancer cells (Figure 3b and c). Our previous reports showed that low energy metabolism and slow proliferation can decrease the susceptibility of cancer cells to anticancer drugs [17,18]. Although upregulated expression of mPEPCK showed the potential to slightly increase the 5-FU susceptibility in both the SNU-C4 and LoVo cell lines, the effects were not statistically significant (Figure 4). Because overexpressed mPEPCK was not found in the mitochondrial fraction (data not shown), further investigations are necessary to validate the effect of subcellular localization of this enzyme on CRT susceptibility.

Although the metabolic characteristics of mPECK remain largely unknown, recent reports suggest that mPEPCK plays various roles in gluconeogenesis and anaplerotic reactions. For example, mPEPCK plays a role in mitochondrial GTP synthesis with insulin release through anaplerotic PEP cycling [19] and cooperates with cytosolic PEPCK to adjust gluconeogenic/TCA flux in response to changes in substrate or energy availability [8]. At present, we cannot explain the molecular mechanism linking mPEPCK expression and susceptibility of CRC cells to 5-FU or radiation. However, expression of both cytosolic PEPCK and mPEPCK may indicate changes in gluconeogenic flux because PEPCK expression can be exquisitely coordinated in parallel with glucose requirements [20]. Our previous reports have shown that the slow energy metabolic process caused by downregulation of key enzymes, such as mitochondrial ATP synthase and pyruvate kinase M2, lead to differential responses of cancer cells to anticancer drugs [17,18]. Thus, mPEPCK downregulation could also lead to slow energy metabolism in CRC cells, and it may subsequently reduce the susceptibility of CRC cells to 5-FU or radiation.

5-FU is known to act as an *S*-phase-specific antimetabolite that suppresses cell proliferation [1,2]. When it enters cells, fluorouracil is converted to 5-fluoro-2′-deoxyuridylate, which can competitively inhibit thymidylate synthase and block the formation of deoxythymidylate, which is required for DNA synthesis [2]. However, the flow cytometry results showed that after 1 μg/ml 5-FU treatment or 8-Gy radiation, the mPEPCK siRNA-transfected cells did not exhibit a significantly different mitochondrial membrane potential or level of apoptosis compared with the NS control (Figure 5a). This finding suggests that downregulation of mPEPCK may affect the energy metabolism of cancer cells, leading to slow proliferation, but that mPEPCK itself cannot augment apoptotic cell death by either 5-FU or radiation treatment. Bad forms a heterodimer with Bcl-2, which is known to induce cells to persist in a G-0 state and to promote cell survival by inhibiting the caspase cascade activation induced by cytochrome c [19,20]. Therefore, in effect, this heterodimer formation counters the antiapoptotic effects of Bcl-2, allowing Bax/Bak-triggered apoptosis [21]. Previous studies have also found that Bax interacts with the permeability transition pore to lower the mitochondrial membrane potential and release cytochrome c, inducing apoptosis [22]. We therefore examined the expression levels of mitochondrial molecules such as Bax, Bcl-2, and Bad in our study. However, in line with the flow cytometry results, the mitochondrial expression levels of Bax, Bcl-2, and Bad were not altered in the mPEPCK siRNA-transfected cells (Figure 5b). To identify a link between mPEPCK and cellular events, we finally investigated the expression of the main cellular signaling pathway molecules, such as AKT, 4EBP1, and mTOR, which are involved in important pathways downstream of Ras (Figure 5c). Lower amounts of p-4EBP1 and p-AKT were detected in the mPEPCK-silenced cells than in the nontransfected SNU-C4 or the NS control (Figure 5c). 4EBP1 plays an important role in the control of protein synthesis, survival, and cell growth [23,24]. When phosphorylated, 4EBP1 has a reduced binding affinity for EIF4E, which leads to the release of EIF4E and the initiation of cap-dependent translation [21]. p-AKT promotes growth factor-mediated cell growth and phosphorylates Bad, hindering apoptosis [22]. Decreased expression levels of p-AKT and p-4EBP1 in the mPEPCK-silenced cells implies that mPEPCK downregulation may not only decrease normal cellular metabolism (Figure 3c), but may be linked to broad cellular events such as abnormal protein synthesis and slow cell proliferation and growth.

To clarify whether our *in vitro* results can be applied clinically, PEPCK expression levels were determined in tissues from patients with rectal cancer who had undergone preoperative CRT. Because of the small amount of tissues available, the mitochondrial fraction could not be isolated from the tissues. However, lower expression levels of total PEPCK (cytosolic and mitochondrial isoforms) in patients’ tumors seemed to be correlated with poorer responses to CRT (Figure 5b). Predicting the tumor response to preoperative CRT can help to tailor treatment plans for locally advanced rectal cancer. PEPCK can be used as a candidate biomarker for predicting the rectal tumor response to CRT. Further studies using standardized measurements and validation will be needed to fully elucidate the potential clinical application of PEPCK as a predictor.

## Conclusions

In conclusion, further studies are needed to elucidate the critical role of PEPCK in 5-FU–based CRT responses. Additionally, a larger sample size is required to confirm the use of PEPCK as a prognostic factor for CRC. However, the present results demonstrate that the slow energy metabolic process caused by downregulation of PEPCK may lead to different responses of CRC cells to 5-FU, radiation, or a combination of the two treatments.

## Competing interests

The authors declare that they have no competing interests.

## Authors’ contributions

JWP, SCK, and BCY participated in the study design. All authors provided study material and were involved in the manuscript writing. All read and approved the final manuscript. JWP, SCK, WKK, and BCY drafted the manuscript.

## Pre-publication history

The pre-publication history for this paper can be accessed here:

http://www.biomedcentral.com/1471-2407/14/160/prepub

## Supplementary Material

Additional file 1: Table S1 PEPCK expression levels in 122 patients with rectal cancer. Click here for file
