# Supplementary material for: Expression of phosphoenolpyruvate carboxykinase linked to chemoradiation susceptibility of human colon cancer cells
Source: BMC Cancer. 2014 Mar 6;14:160. doi: 10.1186/1471-2407-14-160 (PMC4016284; doi:10.1186/1471-2407-14-160)
Supplement: Additional file 1: Table S1 — PEPCK expression levels in 122 patients with rectal cancer. [file 1471-2407-14-160-S1.docx]

**Supporting Information**

**Supplementary Table 1.** PEPCK expression levels in 122 patients with rectal cancer.

*PEPCK expression (PEPCK/Actin) was measured by Western Blot, and the optical densities of the spots were measured from a scanned image (Fig. 6*A*). The PEPCK expression levels of SNU-C4 and HCT-116 were used as controls.

| **WB No. (I-)** | PEPCK | β-actin | PEPCK/Actin* |
| --- | --- | --- | --- |
| 1 | 0.85 | 1.17 | **0.73** |
| 2 | 1.10 | 0.98 | **1.12** |
| 3 | 0 | 1.22 | **0** |
| 4 | 0 | 1.17 | **0** |
| 9 | 1.74 | 1.02 | **1.70** |
| 10 | 0 | 1.09 | **0** |
| 11 | 1.46 | 0.99 | **1.47** |
| 12 | 0 | 0.95 | **0** |
| 17 | 0.96 | 1.23 | **0.78** |
| 18 | 1.07 | 1.33 | **0.81** |
| 19 | 0 | 1.23 | **0** |
| 45 | 0.79 | 1.11 | **0.72** |
| 46 | 0.85 | 1.06 | **0.80** |
| 47 | 0.81 | 1.20 | **0.68** |
| 48 | 0.80 | 1.15 | **0.69** |
| 53 | 0.64 | 1.19 | **0.57** |
| 54 | 1.03 | 1.27 | **0.81** |
| 55 | 0.67 | 1.23 | **0.55** |
| 56 | 0.85 | 1.16 | **0.73** |
| 61 | 1.06 | 1.33 | **0.79** |
| 64 | 0 | 1.28 | **0** |
| 69 | 0.84 | 1.13 | **0.74** |
| 70 | 1.00 | 1.06 | **0.95** |
| 71 | 0.94 | 1.22 | **0.77** |
| 73 | 0.59 | 1.10 | **0.54** |
| 74 | 0.92 | 1.09 | **0.84** |
| 75 | 0 | 1.03 | **0** |

| **WB No. (II-)** | PEPCK | β-actin | PEPCK/Actin* |
| --- | --- | --- | --- |
| 23 | 1.50 | 1.46 | **1.03** |
| 24 | 1.45 | 1.28 | **1.14** |
| 25 | 0 | 1.24 | **0** |
| 26 | 1.21 | 1.34 | **0.90** |
| 31 | 0.77 | 1.39 | **0.55** |
| 32 | 0 | 1.37 | **0** |
| 33 | 1.00 | 1.36 | **0.74** |
| 34 | 0 | 1.35 | **0** |
| 39 | 1.35 | 1.64 | **0.82** |
| 40 | 1.21 | 1.65 | **0.73** |
| 41 | 0.83 | 1.64 | **0.51** |
| 95 | 0.84 | 1.28 | **0.65** |
| 96 | 0.74 | 1.30 | **0.57** |
| 101 | 1.01 | 1.34 | **0.75** |
| 102 | 0.89 | 1.42 | **0.63** |
| 103 | 0.55 | 1.25 | **0.44** |
| 104 | 0.74 | 1.44 | **0.51** |
| 106 | 0.76 | 1.27 | **0.60** |
| 107 | 0.51 | 1.35 | **0.38** |
| 108 | 0.42 | 1.45 | **0.29** |
| 109 | 0.93 | 1.31 | **0.70** |
| 111 | 0.65 | 1.26 | **0.52** |
| 112 | 1.02 | 1.29 | **0.79** |
| 113 | 0.59 | 1.26 | **0.47** |
| 114 | 0.60 | 1.41 | **0.42** |
| 115 | 0.67 | 1.33 | **0.51** |
| 117 | 1.02 | 1.27 | **0.80** |
| 118 | 0.43 | 1.08 | **0.40** |
| 119 | 0 | 1.00 | **0** |
| 120 | 0 | 1.22 | **0** |
| 121 | 1.00 | 1.28 | **0.78** |
| 122 | 1.06 | 1.27 | **0.84** |
| 123 | 0.86 | 1.21 | **0.71** |
| 124 | 0.89 | 1.19 | **0.75** |
| 125 | 0.96 | 1.28 | **0.75** |
| 126 | 0.96 | 1.26 | **0.76** |
| 127 | 1.02 | 1.20 | **0.85** |
| 128 | 0.97 | 1.24 | **0.78** |
| 129 | 0.80 | 1.26 | **0.63** |
| 131 | 0.60 | 1.19 | **0.50** |
| 132 | 0.56 | 1.23 | **0.45** |
| 133 | 1.05 | 1.25 | **0.84** |
| 135 | 1.03 | 1.21 | **0.85** |
| 136 | 0.97 | 1.18 | **0.83** |
| 137 | 1.10 | 1.08 | **1.02** |
| 139 | 0.77 | 1.05 | **0.73** |
| 141 | 0.92 | 1.29 | **0.71** |
| 142 | 0.67 | 0.68 | **0.98** |
| 143 | 0.96 | 1.30 | **0.74** |
| 144 | 0.76 | 1.15 | **0.66** |
| 145 | 0 | 1.17 | **0** |
| 146 | 0.94 | 0.96 | **0.97** |
| 147 | 0.92 | 1.29 | **0.71** |
| 148 | 0.63 | 1.05 | **0.60** |
| 149 | 0.92 | 1.34 | **0.69** |
| 150 | 1.00 | 1.29 | **0.77** |
| 151 | 0.71 | 1.23 | **0.58** |
| 152 | 1.02 | 1.10 | **0.93** |
| 153 | 0.80 | 1.28 | **0.63** |
| 154 | 1.00 | 1.20 | **0.84** |
| 155 | 1.04 | 1.25 | **0.83** |
| 156 | 1.08 | 1.35 | **0.80** |
| 157 | 0.86 | 1.13 | **0.76** |
| 158 | 0.82 | 1.27 | **0.64** |
| 159 | 1.01 | 1.25 | **0.81** |
| 160 | 0 | 1.06 | **0** |
| 161 | 0.73 | 1.28 | **0.57** |
| 162 | 0.68 | 1.26 | **0.54** |
| 163 | 0.87 | 1.26 | **0.69** |
| 164 | 0.87 | 1.26 | **0.69** |
| 165 | 0.46 | 1.18 | **0.39** |
| 166 | 0 | 1.08 | **0** |
| 167 | 0.97 | 1.45 | **0.67** |
| 168 | 1.01 | 1.23 | **0.82** |
| 169 | 1.23 | 1.28 | **0.96** |
| 170 | 0.76 | 1.34 | **0.57** |

| **WB No. (III-)** | PEPCK | β-actin | PEPCK/Actin* |
| --- | --- | --- | --- |
| 27 | 1.42 | 1.29 | **1.11** |
| 28 | 0.68 | 1.24 | **0.55** |
| 29 | 1.28 | 1.19 | **1.08** |
| 30 | 0.93 | 1.19 | **0.78** |
| 35 | 1.38 | 1.36 | **1.01** |
| 36 | 1.62 | 1.48 | **1.10** |
| 37 | 0.74 | 1.48 | **0.50** |
| 38 | 1.37 | 1.30 | **1.06** |
| 42 | 1.27 | 1.78 | **0.71** |
| 43 | 0.98 | 1.57 | **0.62** |
| 44 | 0.89 | 1.42 | **0.63** |
| 45 | 0.96 | 1.38 | **0.70** |

| **WB No. (IV-)** | PEPCK | β-actin | PEPCK/Actin* |
| --- | --- | --- | --- |
| 13 | 1.71 | 0.92 | **1.85** |
| 14 | 1.76 | 0.77 | **2.29** |
| 15 | 1.61 | 0.98 | **1.65** |
| 16 | 1.59 | 0.95 | **1.68** |
| 20 | 1.23 | 1.16 | **1.06** |
| 21 | 0 | 1.13 | **0** |
| 22 | 1.38 | 0.97 | **1.42** |
